# Assessing the Effectiveness of STAPP@Work, a Self-Management Mobile App, in Reducing Work Stress and Preventing Burnout: Single-Case Experimental Design Study

**DOI:** 10.2196/48883

**Published:** 2024-02-29

**Authors:** Sevda Demirel, Yvette Roke, Adriaan W Hoogendoorn, Jamie Hoefakker, Kirsten Hoeberichts, Peter N van Harten

**Affiliations:** 1 Expertise Center for Autism Spectrum Disorder GGz Centraal Almere Netherlands; 2 Department of Psychiatry Amsterdam Universitair Medische Centra Amsterdam Netherlands; 3 Mental Health Program Amsterdam Public Health Research Institute Amsterdam Netherlands; 4 Praktijk InTeam Den Haag Netherlands; 5 Department of Psychiatry GGz Centraal Amersfoort Netherlands; 6 Department of Psychiatry and Neuropsychology School of Mental Health and Neuroscience Maastricht University Maastricht Netherlands

**Keywords:** mental health, stress, coping, burnout, stress management, digital intervention, health promotion, mobile apps, mobile health, mHealth, mental health professionals

## Abstract

**Background:**

Work-related stress and burnout remain common problems among employees, leading to impaired health and higher absenteeism. The use of mobile health apps to promote well-being has grown substantially; however, the impact of such apps on reducing stress and preventing burnout is limited.

**Objective:**

This study aims to assess the effectiveness of STAPP@Work, a mobile-based stress management intervention, on perceived stress, coping self-efficacy, and the level of burnout among mental health employees.

**Methods:**

The study used a single-case experimental design to examine the use of STAPP@Work among mental health employees without a known diagnosis of burnout (N=63). Participants used the app for 1 week per month repeatedly for a period of 6 months. Using a reversal design, the participants used the app 6 times to assess replicated immediate (1 week after use) and lasting (3 weeks after use) effects. The Perceived Stress Scale, the Coping Self-Efficacy Scale, and the Burnout Assessment Tool were used to measure the outcomes. Linear mixed models were used to analyze the data.

**Results:**

After 6 months of app use for 1 week per month, the participants showed a statistically significant decrease in perceived stress (b=–0.38, 95% CI –0.67 to –0.09; *P*=.01; Cohen *d*=0.50) and burnout symptoms (b=–0.31, 95% CI –0.51 to –0.12; *P*=.002; Cohen *d*=0.63) as well as a statistically significant improvement in problem-focused coping self-efficacy (b=0.42, 95% CI 0-0.85; *P*=.049; Cohen *d*=0.42). Long-term use of the app provided consistent reductions in burnout symptoms over time, including in the level of exhaustion and emotional impairment.

**Conclusions:**

The use of an app-based stress management intervention has been shown to reduce burnout symptoms and enhance coping self-efficacy among mental health workers. Prevention of burnout and minimization of work-related stress are of utmost importance to protect employee health and reduce absenteeism.

## Introduction

### Background

Work-related stress remains a persistent challenge for both employees and organizations, adversely affecting health outcomes and reducing job performance [1]. Stress is often defined as a condition in which an environmental demand exceeds the natural regulatory capacity of an organism, particularly in situations that are unpredictable and uncontrollable [2]. Numerous studies over the last several decades have demonstrated that psychosocial stress adversely affects hormonal processes and organ systems in the body [3-6]. Excessive stress has a detrimental effect on mental health by causing emotional distress and increasing the risk of mental illnesses such as depression, anxiety, and burnout [7].

Burnout is defined as a psychological syndrome primarily caused by a prolonged period of stress and the inability or unwillingness to cope effectively, resulting in both physical and mental exhaustion [8]. The term burnout is often used in the occupational context, where stress originates from the work environment. Such job stressors include heavy workload, long working hours, job insecurity, conflicts, low support, role ambiguity, and a lack of autonomy [9]. When work stress is of a long duration without applying effective coping strategies, job demands exceed capacity, leading to the depletion of one’s emotional, physical, and cognitive resources [9]. Individual consequences further include job dissatisfaction, depersonalization, cynicism, and low fulfillment [10]. Organizational consequences include absenteeism, reduced productivity levels, a higher turnover rate, and an increase in health care costs [9,11]. Organizations may incur financial costs not only from losing and replacing experienced staff but also by providing employee burnout support, implementing employee assistance programs, and facilitating reintegration processes [11-14]. Burnout causes significant distress owing to symptoms such as chronic fatigue, trouble sleeping, depressive symptoms, and difficulties with concentration and memory [9]. In 2021, approximately 17% of Dutch employees reported being mentally fatigued by their work, 16% were emotionally exhausted, and 15% were completely exhausted after work [15]. Moreover, the Dutch health care sector had one of the highest proportions of employees experiencing work-related mental exhaustion, at 20% [16].

Effective interventions for preventing work-related stress and burnout are of high importance. The use of digital health, including mobile apps, as behavioral interventions to promote positive mental health is rapidly growing. The benefits of mobile mental health interventions include, but are not limited to, high accessibility, low costs, less time consumption, no clinician involvement, timely support, and the promotion of autonomy [17]. A recent systematic review found that mindfulness was the most commonly applied theory-based stress management strategy in apps [18]. They further concluded that such apps implemented stress prevention strategies less often than stress management strategies for stress recovery [18]. However, stressor identification and reduction, as primary prevention, is a crucial element in effective stress management [19]. Moreover, another review found that only a few stress management apps combined both stress monitoring and intervention based on detecting users’ stress levels [20].

Previous studies examining the effectiveness of web- and app-based interventions among employees provided evidence of their effectiveness in stress reduction [21]. Improvements regarding emotional exhaustion and perceived stress in health care professionals using digital interventions were reported earlier as well [22,23]. However, the few available studies on apps for health care workers yielded mixed findings on stress and were often based on mindfulness practices or psychoeducation. For instance, 2 randomized controlled trials (RCTs) assessing an app among health care workers found no significant improvements in stress [24,25]. Although other mindfulness-based apps did find improvements in stress among physicians, nurses, and therapists [26]; hospital nurses [27]; and employees within the general practitioner practice [28], little is known regarding the effectiveness of app-based interventions aimed at stress management and burnout symptoms among employees, specifically health care professionals. Moreover, there is a lack of evidence for the sustained effects of long-term use of such app-based interventions [29].

Mobile-based ecological momentary interventions (EMIs) comprise an emerging area of research. These interventions are based on ecological momentary assessment (EMA), a widely used tool in psychiatry to collect repeated daily assessments of individuals’ behaviors, emotions, experiences, or thoughts as they occur in their own “real-world” settings [30]. EMA is seen as a promising method that provides insight into variations in symptoms and behavior over time and their underlying person-environment interactions. EMIs offer interventions in the moment of an individual’s daily life, usually based on the assessment [31]. Previous studies on the effects of EMIs showed a positive overall medium effect on stress in clinical populations [32]. To date, no study has assessed the effectiveness of a mobile stress management intervention based on stress monitoring among health care workers.

### Objectives

To bridge this gap, our group developed a self-management stress-signaling app using a participatory design with mental health employees called STAPP@Work. The app is developed to monitor daily stress levels and track the daily activities in employees’ natural settings, including work settings, based on the principles of EMA. STAPP@Work is a tool to manage stress at work by providing the employee feedback regarding their tracked stress levels and associated activities and creating a visual overview to make stress patterns during workdays and weeks visible. Furthermore, the app provides real-time suggestions for coping strategies specified for both the work environment and at home. The aim of this study was to evaluate the effectiveness of the STAPP@Work app by examining perceived stress, coping self-efficacy, and burnout symptoms. On the basis of previous literature outlined in the *Background* section, we hypothesized that after 6 months of using the STAPP@Work app for 1 week per month, perceived stress and coping self-efficacy would be improved and burnout symptoms would be reduced in mental health workers. In addition, we explored the progression of perceived stress, coping self-efficacy, and burnout scores over time to examine their lasting effects.

## Methods

### Study Design

This study adopted a single-case experimental design (SCED), a within-person design that measures the effectiveness of interventions [33]. Using an SCED, it became possible to focus on individual changes in outcomes (perceived stress, coping self-efficacy, and burnout symptoms) over time. This design was considered a good fit, as it enabled the assessment of behavior change over time in “real-world” settings using heterogeneous samples [33]. Moreover, SCEDs are often used to capture small but meaningful differences in behavior and to examine behavior that may change gradually over time, which is not feasible to assess in RCTs with a high number of successive measurements [34]. In addition, SCEDs are suitable for being able to evaluate the effectiveness of novel innovative interventions before they are evaluated in time-consuming and costly RCTs [35]. The results of this SCED study will provide an indication of whether it is worthwhile to conduct a large-scale RCT to further assess the effectiveness of the app [36]. Given that STAPP@Work is a novel behavioral intervention, using an SCED constitutes a useful first step to measure its effectiveness.

Furthermore, an A1-B1-A2-B2 reversal design was used, in which A1 represents the baseline period, B1 the introduction of an intervention, A2 the withdrawal of the intervention, and B2 the reintroduction of the intervention [36]. In this study, this translated to the participants having used the STAPP@Work app for 1 week after the baseline period, followed by 3 weeks of nonuse, after which they used the app again for the second time for a weeklong period. Withdrawing the intervention allowed for an assessment of whether the effects of the app were lasting.

### Sample Size

Owing to the complexity and practical unfeasibility of the sample size calculation for this SCED, the sample size for this study was based on the traditional paired sample 2-tailed *t* test design. Similar studies on stress management apps among health care employees are limited. Instead, we relied on a meta-analysis of the effectiveness of web- and app-based interventions on stress among employees, which found moderate-to-large effect sizes for mindfulness-based interventions and small-to-moderate effect sizes for stress management interventions [37]. A medium effect size (Cohen *d*=0.5) was considered in the sample size calculation for this study. To conduct a paired sample 2-tailed *t* test and detect a medium effect size (Cohen *d*=0.5) with a low internal correlation (*r*=0.1), a sample size of 59 participants was required. Considering the potential dropout rate of 7.5%, the required sample size was set at 63 participants.

### Participants

Participants were eligible to participate in the study when they were employed within GGz Centraal, the fourth largest mental health care institution in the Netherlands. No further inclusion or exclusion criteria were applied. Recruitment strategies included the promotion of the study through flyers, digital media, and approaching teams or staff in various departments.

### The STAPP@Work App

#### App Overview

STAPP@Work is a self-management app designed to manage stress and improve well-being by (1) measuring daily stress levels, (2) providing personal coping advice, and (3) visualizing stress patterns. The app is based on a stress-signaling plan, a widely used tool in Dutch mental health institutions to monitor clients’ stress levels in relation to their mental health [38]. This allows for recognizing and dealing with early warning signs of distress. A stress-signaling plan often consists of 4 phases: green, yellow, orange, and red. Each phase characterizes how a person is feeling and what symptoms are associated, with the colors increasing from green (no stress) to red (very high stress) [38]. Furthermore, the plan describes both stress-inducing and protective factors as well as personal coping strategies to encourage the clients’ self-management [38]. STAPP@Work includes the concept of a stress-signaling plan and the EMA method by assessing stress levels in daily life and ensuring that these are linked to specific moments and activities in a day. The app was designed together with mental health employees and was based on their needs, ideas, and experiences that were explored during focus group discussions. The ongoing iterative development of the prototypes continued until the desired version of the STAPP@Work app was achieved.

#### Measuring Daily Stress

**Figure 1:**
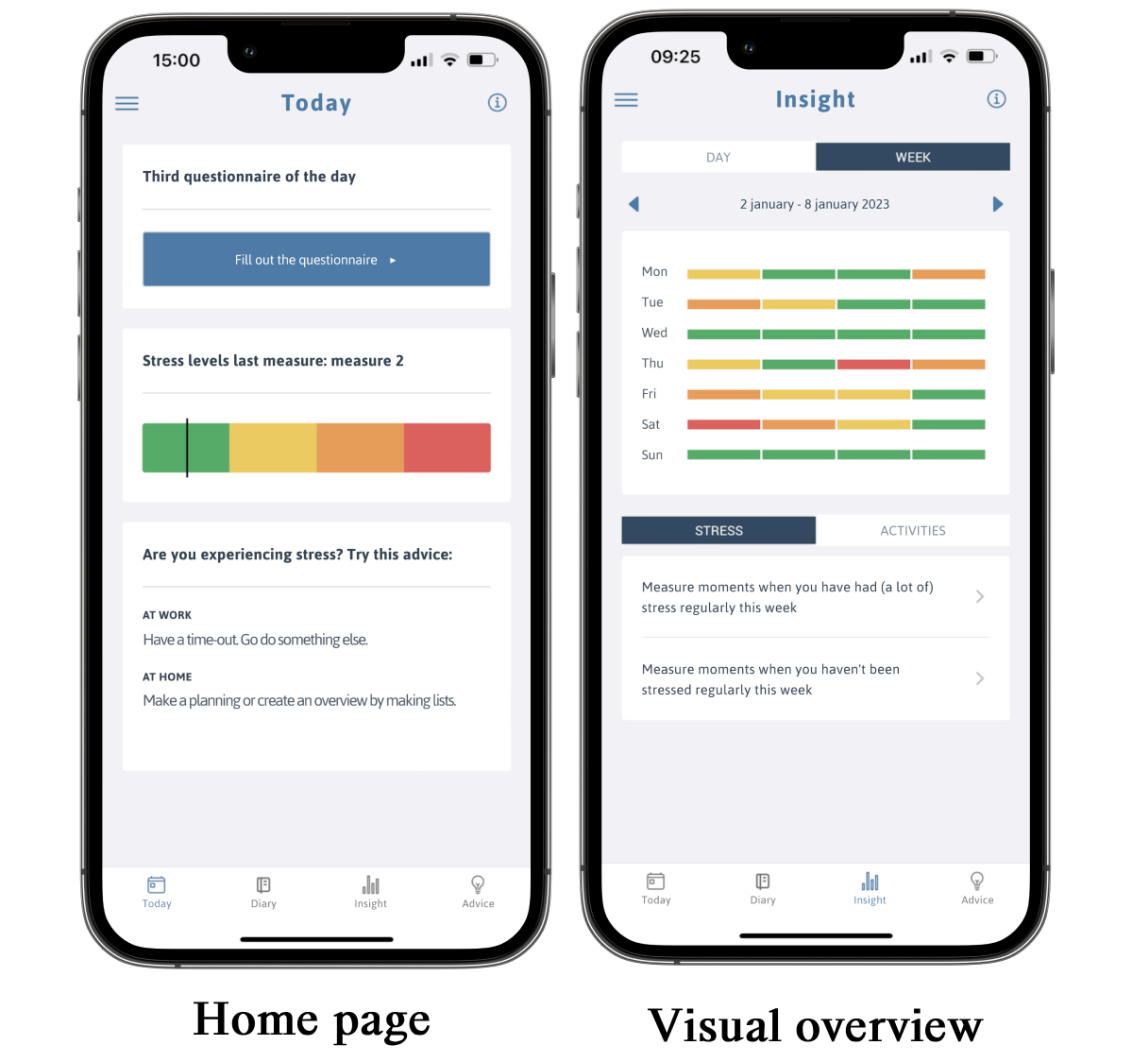
Screenshots of the home page and the visual overview page of the STAPP@Work app (translated from Dutch).

The app calculated daily stress levels by preparing a questionnaire. The employee could specify whether to complete 2, 3, or 4 questionnaires per day via the app settings. The app required a minimum of 2 questionnaires, as there were 4 hours between each questionnaire, and filling in 2 questionnaires would cover a working day of 8 hours. If the employee chose to fill in 3 or 4 questionnaires a day, the app would cover a 12-hour or 16-hour period out of 1 day. On the home page, the user could see if a questionnaire was available to fill in as well as the results of the previous stress measurement, as shown in Figure 1.

Each questionnaire became available for the user to complete within a 1-hour time frame, probing activities that were performed in the past 4 hours and how one felt while doing so. Then, it followed with 7 stress-signaling questions (eg, “Did you feel irritable?” and “Were you able to take good care of yourself?”) to derive a stress level. The app calculates stress levels based on the answers to 7 stress questions. The stress questions were formulated and validated by focus group discussions and individual interviews with employees, following the design thinking method. The questions were not derived from an existing stress scale. The answer options for each stress question are as follows: “no”; “yes, but not more than normal”; “yes, more than normal”; and “yes, much more than normal.” A score is assigned to each answer option, and from there, the app computes a total stress score based on the answers to all 7 stress questions. The app then assigns the user’s perceived stress to 1 of 4 categories: no stress, little stress, stress, or a lot of stress, based on the total score from the stress-related questions. The app also asked for the user’s feedback on the assigned stress categories and gave personal coping advice.

#### Visual Overview

The app includes a visual overview of the assigned stress levels at both the day and week levels, helping the users to recognize their own stress patterns (Figure 1). The overview also showed which activities were associated with (a lot of) stress and no stress during the week.

#### Personal Coping Advice

During the exploratory focus groups and interviews, employees were also asked how they cope with stress at work and at home. Together with desk research and practitioner experience, a list of different effective approaches to coping with stress was formed, which was referred to as coping strategies. This list forms the general list of predefined suggestions for coping in the app. Suggestions for coping at work included the following: contact or discuss the situation with a colleague, get moving, go walking, plan or make an overview, do breathing exercises, take a break, do something else, and take care of yourself such as eating and drinking enough. However, the participants could add their own suggestions for coping to the list and remove existing suggestions. After each stress assessment, the app suggests a coping strategy displayed in text, which the app randomly selects from the personalized coping list of the user.

#### Personalization

The app was highly customizable, as the users could choose how many questionnaires they wanted to fill out in a day and at what times, to fit their working days. The app consisted of a list of activities created by mental health employees that fit within a workday, but users could add or remove activities as they wished. The same was true for coping advice, in which the app provided a list of coping strategies that the user could change at any time.

### Measures

#### Perceived Stress

The Perceived Stress Scale (PSS) is a 10-item self-report questionnaire that was used to measure how stressful certain situations were perceived in the past month (eg, “How often have you felt that you were in control of things?”), with items rated on a 5-point scale ranging from 0 (never) to 4 (very often) [39]. The PSS has shown good internal consistency (10 items; Cronbach α=0.84) [40].

#### Coping Self-Efficacy

The 13-item version of the Coping Self-Efficacy Scale (CSES) measures one’s confidence in various coping behaviors [41]. The CSES consists of 3 subscales: problem-focused coping (6 items; Cronbach α=0.91), emotion-focused coping (4 items; Cronbach α=0.91), and social support coping (3 items; Cronbach α=0.80) [41]. Items have a 10-point scale ranging from 0 (cannot do at all) to 10 (certainly can do).

#### Burnout Symptoms

The shorter version of the Burnout Assessment Tool (BAT), which consists of a 12-item self-report questionnaire, determines the extent to which burnout symptoms are experienced (eg, “I do not recognize myself in the way I react emotionally at work”), rated from 0 (never) to 4 (always) [42]. Cronbach α has been shown to be similar to the full version of the BAT (23 items) [43]. The BAT (23 items; Cronbach α=0.90) is subdivided into 4 validated core dimensions: exhaustion (8 items; Cronbach α=0.92), mental distance (5 items; Cronbach α=0.91), cognitive impairment (5 items; Cronbach α=0.92), and emotional impairment (5 items; Cronbach α=0.90) [42].

### Procedure

A measurement involved filling out the questionnaire that was composed of the PSS, CSES, and BAT items. The researchers (KH and JH) sent the web-based questionnaire to all participants at each time of measurement, regardless of whether they had completed the previous questionnaire or not. A total of 17 measurements were obtained from the participants over a period of 9 months. Of these, 24% (4/17) were conducted during the baseline period and 71% (12/17) during the intervention phase, ending with 6% (1/17) follow-up measurement. We chose to include 4 baseline measurements to account for possible learning effects from merely participating in the study. Being introduced to the study and responding to the questionnaires on stress could have possibly contributed to an increased awareness of one’s own stress. During the 7-week baseline phase, the participants completed the questionnaire every 2 weeks. After the baseline measurement, the research team provided individual explanatory sessions on installing, setting up, and using the STAPP@Work app as well as the proceedings of the experiment. The participants could share their comments and questions about the study, as well as inquire about app functionalities, with the help desk that was available.

**Figure 2:**
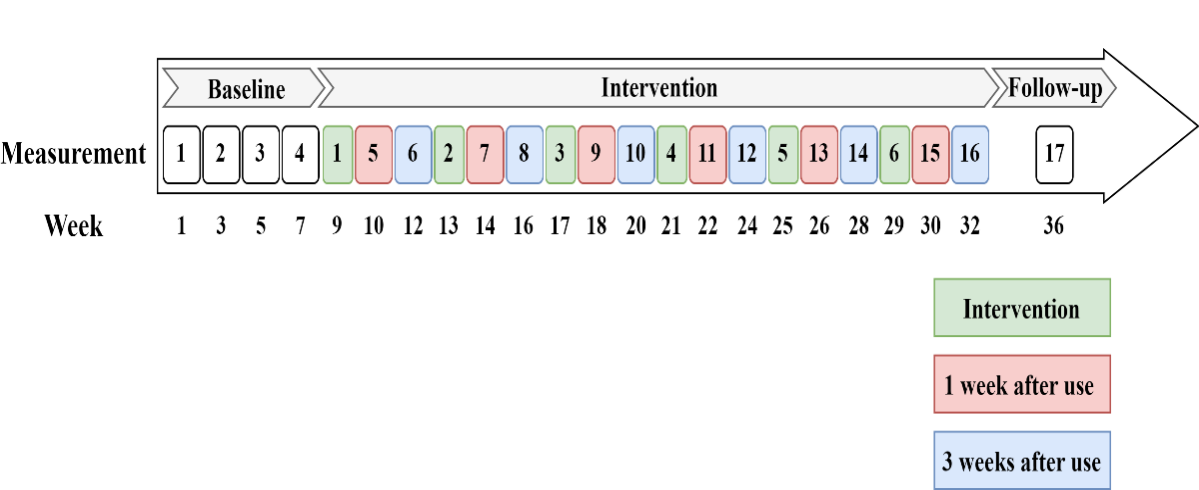
Schematic overview of the study design, including the timeline.

The intervention concerned the daily use of the STAPP@Work app for 1 week, called the intervention week. After the withdrawal of the intervention, a questionnaire was completed in the week after use (1 week after use) and 3 weeks after use to examine the immediate and lasting effects, respectively. The introduction and withdrawal of the intervention were repeated 6 times throughout the intervention phase. A follow-up measurement took place 6 weeks after the last time the app was used. Figure 2 shows the order of the events.

### Statistical Analysis

All analyses were performed using SPSS (version 28; IBM Corp). For data analysis, mean scores were calculated from the total scores of the PSS, CSES, and BAT, including the subscales. The subscales of the CSES and BAT were included as separate outcome variables to gain more insight into the subdomains. Descriptive statistics were used to describe participant characteristics and determine response rates over time. We categorized participants as early dropouts if they failed to use the app at least 3 times and definitively ceased participation. Data analysis did not include early dropouts. Linear mixed model (LMM) analysis was used to analyze the data [44]. LMMs are well suited for longitudinal intervention studies, as they allow the measurement of change in the response variables across units of time and within participants [45,46] and account for missing values [47]. Change scores between baseline and all consecutive intervention measurements were modeled for all outcome variables. We chose the fourth and last baseline measurement (week 7) as a reference to correct for learning effects during the baseline period [36].

When conducting the LMM, assumptions of normality and equal variance of residuals were checked using graphical representations. As the data were collected over time, the presence of autocorrelation was evaluated both visually by the partial autocorrelation function plot and by the Durbin-Watson statistic test. Accordingly, the appropriate covariance structure was selected for the models for each outcome variable. Time was represented by (binary) week indicators that were included in the LMM as fixed effects, enabling the assessment of mean scores for each week of app use. For each outcome, Cohen *d* effect sizes were calculated for the mean change score from baseline to 6 months, standardized by the baseline SD. The LMM results were used to create graphs, visually presenting the estimated mean scores of the outcomes and illustrating the progression over time. The significance level was set to .05.

### Ethical Considerations

To ensure data confidentiality, consideration has been given to secure data collection and storage. Data were collected through web-based questionnaires and stored in a folder. Only researchers (JH, KH, YR, and SD) within the team with access could view the responses. Data were then anonymized and stored in a protected folder on a secure server of GGz Centraal, where access for a single researcher (SD) was protected by a password and a soft token-based authentication. This study was conducted in accordance with the Declaration of Helsinki and was approved by the Medical Ethics Committee of Isala Zwolle (211006211006) on November 11, 2021. Informed consent was obtained from all participants involved in the study. The informed consent form included extensive information regarding the purpose of the study, procedures, data security, and the participant’s rights. Participation in the study was completely voluntary and without any compensation. Furthermore, the use of the STAPP@Work app is in accordance with the General Data Protection Regulation of the European Union.

## Results

### Sample Characteristics

A total of 63 mental health employees were included in the study, with 10 early dropouts, resulting in 53 (84%) participants. Of these, 74% (39/53) were female participants, and the average age was 41 (SD 11.7) years (Table 1).

Various mental health professions were included, with the majority represented by nurses (22/53, 42%). The response rates of the participants ranged from 45% (24/53) to 100% (53/53), as presented in Table 2.

**Table 1:** Characteristics of the participants (n=53).

Characteristic			Value
Age (years), mean (SD)			41 (11.7)
**Sex n (%)**			
	Female	39 (74)	
	Male	14 (26)	
**Profession, n (%)^a^**			
	Nurse	22 (42)	
	Psychologist	7 (13)	
	Management function	7 (13)	
	Counselor	7 (13)	
	Staff member or adviser	4 (8)	
	Administration or IT function	3 (6)	
	Psychiatrist	2 (4)	
	Medical doctor in training	1 (2)	

^a^Due to rounding, percentages may not add up to 100%.

**Table 2:** Response rates among participants (n=53).

	Questionnaire	Value, response rate (%)^a^
Baseline	Questionnaire 1	53 (100)
Baseline	Questionnaire 2	52 (98)
Baseline	Questionnaire 3	52 (98)
Baseline	Questionnaire 4	51 (96)
Intervention	Questionnaire 5	46 (87)
Intervention	Questionnaire 6	42 (79)
Intervention	Questionnaire 7	41 (77)
Intervention	Questionnaire 8	41 (77)
Intervention	Questionnaire 9	37 (70)
Intervention	Questionnaire 10	38 (72)
Intervention	Questionnaire 11	35 (66)
Intervention	Questionnaire 12	29 (55)
Intervention	Questionnaire 13	24 (45)
Intervention	Questionnaire 14	27 (51)
Intervention	Questionnaire 15	28 (53)
Intervention	Questionnaire 16	26 (49)
Follow-up	Questionnaire 17	30 (57)

^a^Response rate refers to the number of participants who completed the questionnaire.

### Perceived Stress

The perceived stress score significantly improved after 6 months (b=–0.38, 95% CI –0.67 to –.09; *P*=.01) compared with baseline, with a medium effect size (Cohen *d*=0.50), as presented in Table 3.

**Figure 3:**
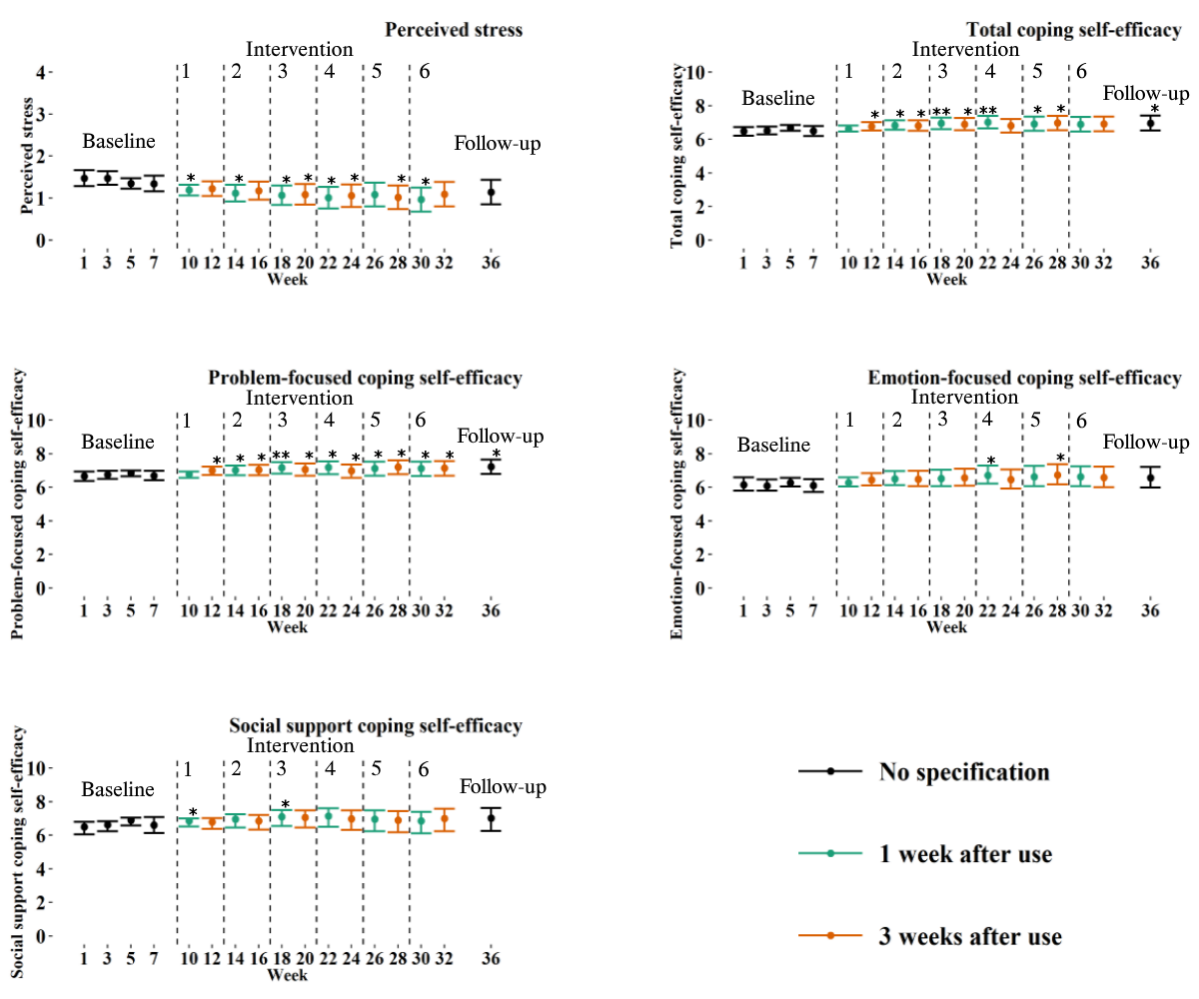
Graphs showing the progression of the estimated means from linear mixed models of perceived stress, total coping self-efficacy, problem-focused coping self-efficacy, emotion-focused coping self-efficacy, and social support coping self-efficacy over a 36-week period. The baseline, intervention, and follow-up phases are presented. Each intervention week is indicated by the corresponding number. Error bars represent CIs. *P<.05. **P<.01 significant change from baseline (wk 7).

Figure 3 shows a lower stress score after each intervention week for 1 week after use, except for the fifth intervention week. A significant decrease in stress score was not found at follow-up.

**Table 3:** Results of linear mixed model for all outcome variables: perceived stress, coping self-efficacy with subscales, and burnout symptoms with subscales after 6 months of monthly 1-week app use.

Variable			Unstandardized beta coefficient, b (SE: 95% CI)		*P* value		Cohen *d*
Perceived stress			–0.38 (–0.15; 0.67 to –0.09)		.01		0.50
**Total coping self-efficacy^a^**			0.40 (0.22; –0.03 to 0.83)		.07		0.37
	Problem-focused coping	0.42 (0.21; 0 to 0.85)		.049		0.42	
	Emotion-focused coping	0.52 (0.30; –0.08 to 1.11)		.09		0.17	
	Social support coping	0.24 (0.33; –0.41 to 0.89)		.47		0.29	
**Total burnout symptoms^b^**			–0.31 (0.10; –0.51 to –0.12)		.002		0.63
	Exhaustion	–0.38 (0.15; –0.68 to –0.08)		.02		0.50	
	Mental distance	–0.22 (0.14; –0.48 to 0.05)		.11		0.34	
	Emotional impairment	–0.42 (0.13; –0.68 to –0.16)		.002		0.69	
	Cognitive impairment	–0.24 (0.13; –0.49 to 0.01)		.06		0.41	

^a^Total score of all 13 items on the Coping Self-Efficacy Scale.

^b^Total scores of all 12 items on the Burnout Assessment Tool.

### Coping Self-Efficacy

Coping self-efficacy did not significantly improve after 6 months of app use for 1 week per month (b=0.40, 95% CI –0.03 to 0.83; *P*=.07), as shown in Table 3. Focusing on the subscales, problem-focused coping abilities significantly improved after 6 months (b=0.42, 95% CI 0-0.85; *P*=.049) with a small to medium effect size (Cohen *d*=0.42), and emotion-focused coping (b=0.52, 95% CI –0.08 to 1.11; *P*=.09) and social support coping (b=0.24, 95% CI –0.41 to 0.89; *P*=.47) were not significantly improved. Figure 3 shows significant, consistent improvements in problem-focused coping starting from the second intervention week for both 1 week and 3 weeks after use until follow-up. No significant, consistent improvements were found regarding emotion-focused and social support coping self-efficacy scores (Figure 3).

### Burnout Symptoms

Table 3 shows a significant improvement in the measure of burnout symptoms after 6 months of app use for 1 week per month (b=–0.31, 95% CI –0.51 to –0.12; *P*=.002) compared with baseline, with a medium effect size (Cohen *d*=0.63). Regarding the subscales, a significant decrease was found for exhaustion (b=–0.38, 95% CI –0.68 to –0.08; *P*=.02) and emotional impairment (b=–0.42, 95% CI –0.68 to –0.16; *P*=.002). The effect sizes were found to be medium for both exhaustion (Cohen *d*=0.50) and emotional impairment (Cohen *d*=0.69).

**Figure 4:**
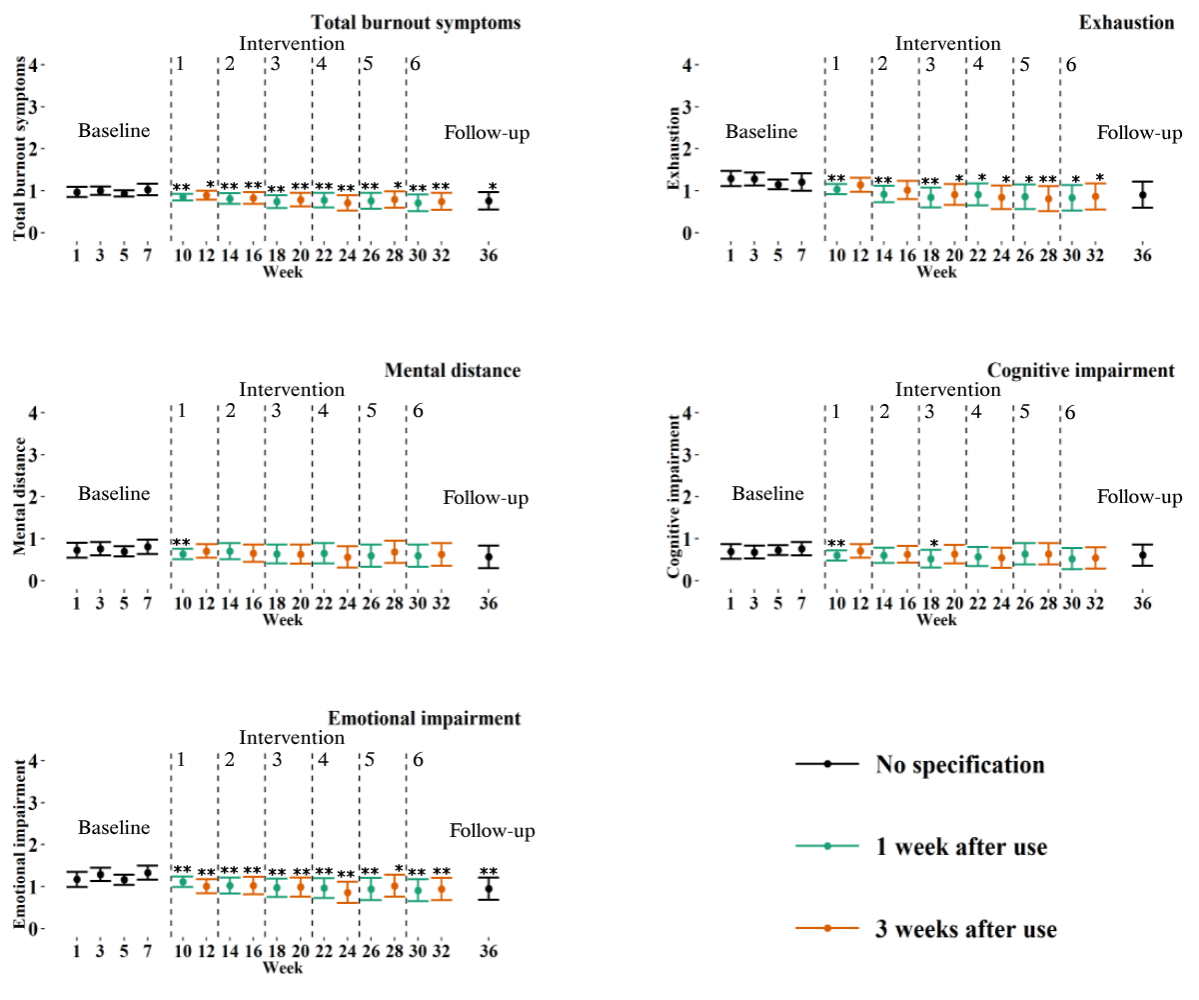
Graphs showing the progression of the estimated means from linear mixed models of total burnout symptoms, exhaustion, mental distance, cognitive impairment, and emotional impairment over a 36-week period. The baseline, intervention, and follow-up phases are presented. Each intervention week is indicated by the corresponding number. Error bars represent CIs. *P<.05, **P<.01 significant change from baseline (wk 7).

When examining the progression over time, a significant decrease in emotional impairment was found after the first week of app use and was sustained for both 1 week post use and 3 weeks after use until follow-up (Figure 4). Consistent improvements following initial app use were found for exhaustion as well, with no significant change at follow-up (Figure 4). No consistent and sustained improvements were found for the level of mental distance and cognitive impairment (Figure 4).

## Discussion

### Principal Findings

This effectiveness study showed that the participants using STAPP@Work were able to consistently reduce their measure of burnout symptoms over time. Specifically, the findings consistently showed improvements in exhaustion and emotional impairment, with sustained improvement observed in emotional impairment. Simultaneously, the participants showed lasting improvements in their confidence in problem-focused coping abilities. Perceived stress levels were reduced after 6 months of monthly 1-week app use. Following the findings, the app provides a means for improving problem-focused coping strategies to cope effectively with job stressors and reduce burnout complaints.

This study found lasting improvements in burnout symptoms. Although each participant scored a measure of burnout symptoms, it is important to emphasize that they did not have a known burnout diagnosis. All participants were working during the time of this study. From the findings, it became apparent that particularly 2 core dimensions—exhaustion and emotional impairment—were consistently reduced. Improvements in exhaustion indicate that the employees felt less tired and experienced higher physical and mental energy levels at work [42]. A decrease in emotional impairment implies that the participants were able to improve their abilities to adequately control their own emotions at work [42]. The app providing insights into one’s stress patterns, and work-related stressors may have contributed to higher awareness and control over one’s stress levels at work. The results of this study showed reductions in perceived stress among the employees. The employees may have been able to reduce their stress as the app assessed their perceived stress several times a day, allowing them to intervene early when stress levels were rising to prevent worsening, similar to how a stress-signaling plan functions [38].

Moreover, they improved their problem-focused coping self-efficacy, indicating increased confidence in coping effectively by resolving the stressful situation or altering the source of the stress [41]. This suggests that the participants were more able to cope effectively with work-related stressors and demands, resulting in lower exhaustion and higher control of emotional processes. Stress assessment and surveillance are crucial for determining the causal and contributing factors of stress according to the theory of preventive stress management (PSM) [19]. The PSM framework further encourages individual stress assessment by assuming a transactional relationship between an individual and work-related stress because of individual differences [19]. STAPP@Work calculated daily stress levels and created a personal overview in which one could see the moments and activities that were connected to high stress. This allowed the employee to recognize moments of high stress during the day and week as well as the associated activities to identify stress-inducing factors. This may have empowered employees to lessen, modify, or manage recognized work stressors, aiming to reduce or prevent the stress response as part of primary prevention [19]. Addressing work stressors reduces high job demands, leading to less fatigue and more energy, as the job demands–resources model assumes that high job demands and low resources are important predictors of the exhaustion component of burnout [48].

The PSM theory argues that chronic stress that remains unaddressed and builds over time is manageable by providing interventions focused on both primary and secondary prevention [19]. The app addresses secondary prevention by providing real-time suggestions for coping strategies to manage work stress. Stressful situations become more manageable and less threatening because of such effective coping strategies, which act as a protective factor for the development of burnout [9]. Improvements in problem-focused coping self-efficacy were both immediate and lasting, suggesting that app use has led to the learning of problem-focused coping skills. In contrast, emotion-focused and social support coping abilities did not significantly improve; therefore, our hypothesis only supports problem-focused coping self-efficacy. The features of the app may have been especially profitable for problem-focused coping. Insights into stress patterns and daily stress levels through app use may have caused participants to be more proactive in identifying and resolving the cause of stress. Previous research has shown that individuals engaged more in problem-focused coping instead of emotion-focused coping when they were clearer and more communicative about their own emotions [49]. Therefore, participants gaining insights and self-reflection regarding their stress level may have little to gain by engaging in emotion-focused coping.

Indeed, the perceived stress level significantly reduced after 6 months; however, when examining the effects over time, these were often to be found immediate instead of lasting with no significant change at follow-up. This implies that the app is most effective when users actively engage with it, leading to increased awareness and control. Conversely, when the app is not in use and does not provide feedback on stress levels, the effects diminish until the app is used again.

The few studies examining mental health apps in relation to burnout have shown similar results in the improvement of burnout symptoms among employees [50] and specifically in reducing emotional exhaustion among health care professionals [23,51]. Furthermore, findings from an RCT assessing a self-management app among employees showed medium effects for reduction in perceived stress, lower emotional exhaustion, and higher emotion regulation, which are consistent with our findings [52]. Most of the scientifically studied mental health apps were based on the theories of mindfulness, psychoeducation, or cognitive behavioral therapy and were studied in RCTs [22,53]. The findings of this study add to the existing body of research by providing evidence that a stress management app, aligned to the work environment and assessed in a naturalistic context, can reduce symptoms that indicate burnout and, therefore, has the potential to preventively avert the risk of burnout. Furthermore, this study provides insights into the sustained effects of mental health apps, which were often lacking in previous studies [29].

### Strengths and Limitations

One of the strengths of this study includes the use of an SCED, which made it possible to offer the intervention to all participants [20] and enabled the measurement of the changes of each participant in “real-life” conditions rather than comparing them between the 2 groups in a highly controlled setting [54]. Furthermore, the relatively long length of the experiment allowed for close observation of how stress, coping, and burnout symptoms progressed separately over a 9-month period and whether these were learned over time, such as coping [33]. In addition, STAPP@Work involves an innovative design in which an app incorporates a stress-signaling intervention and is of potential value in terms of reducing absenteeism.

There are several limitations to consider when interpreting this study. First, the participants may have induced a social desirability bias. However, it is unlikely that such a bias persisted for several months after the intervention. Second, we observed that the number of missing values increased as the study progressed. Participants may have failed to adhere because of a loss of interest or other external circumstances. However, LMMs can deal with dropout if the missing data mechanism is missing at random, which is the case if dropout depends on observed characteristics such as stress in the baseline period [47].

Third, each participant received the intervention at the same time following the baseline period, as it was not feasible to measure baseline stability at the individual level. Such stability is valuable to ensure that results are accountable to the intervention rather than to contextual influences [55]. This study did apply a reversal design, contributing to a higher degree of experimental control by repeatedly introducing and withdrawing the intervention, leading to a more robust demonstration of replicated effects and resulting in higher internal validity [56]. Fourth, the small sample size and disproportionate representation of female participants limited the generalizability of the findings to a broader and more diverse population. However, the distribution of sex in this study population corresponds to the distributions reported for Dutch mental health institutions [57]. Moreover, potential knowledge on stress management by mental health professionals restricts the generalizability to other industries or work settings as well. Finally, as this study followed a repeated measures design, it became more difficult to assess if changes occurring over time were a result of repeated use of the app or rather the cumulative effects of previous app use [36]. However, this did not limit the interpretation of whether such previous effects of the app were lasting.

### Implications and Future Research

The preliminary results provided promising evidence that STAPP@Work is effective in reducing burnout symptoms and improving problem-focused coping. These findings build upon existing research by showing that a mobile health app can lead to sustainable effects and is able to address burnout-related symptoms. This is particularly relevant for preventive implementation in organizations to prevent burnout. This self-management app may have the potential to offer support in occupational contexts to promote less absenteeism and a healthier workforce, which are important for well-functioning and successful organizations. In addition, the app has the potential to integrate into daily life to support employees during their workdays or challenging periods. As the app helps to identify job stressors and provides coping advice suited for work environments, it can be implemented in any workplace to protect the health of employees and enhance job satisfaction.

Because of their high accessibility and autonomy, mobile interventions can be a more convenient and resource-saving way to prevent the onset of aggravated stress symptoms from within the workplace. This is especially relevant as recent challenges in the health care workforce include high-pressure working conditions and a shortage of workers, although the number of employees has increased in recent years [58]. It is of utmost importance to focus on the health of these employees, who are putting in the effort to provide the best care for clients.

An SCED study is typically characterized by a within-person design. Hence, this design does not include a control group but instead uses longer baseline periods and multiple measures over time. This impeded the ability to draw firm conclusions on the effects of the app, especially for a larger group, and therefore highlights the need for future experimentally controlled studies investigating larger and more heterogeneous samples. Further qualitative research is necessary to complement the findings of this study, as it remains unclear how the app contributes to perceived stress, coping skills, and burnout symptoms. By exploring this context and examining the underlying mechanisms and motivations for employees’ behavior, a richer understanding of users’ experiences and more insight into the meaning of the app in everyday work life are created [59,60].

Although the intervention resulted in a significant reduction in perceived stress immediately after using STAPP@Work, this study found no significant change in stress levels at follow-up. This suggests that the effectiveness of the app in reducing stress in the short term did not persist in the long term. This underscores the importance of conducting future research studies to investigate this specific aspect further. In addition, it would be beneficial to conduct further research on longer-term follow-up assessments to gain more insight into the lasting impact of the app. As the app shows promise in reducing burnout symptoms, it would be clinically relevant to explore the effect of the app on specific groups of individuals, for instance, those with a history of burnout or absenteeism. Because of the accessibility of the app, it can offer solutions to those individuals who are in immediate need of support in managing their stress, such as clients who are on the waiting list for psychological help, which prompts further research ideas.

### Conclusions

This intervention study showed that STAPP@Work, as a stress management app, is effective in reducing burnout symptoms and improving problem-focused coping skills among mental health workers. The preliminary findings specifically showed reductions in exhaustion levels and emotional impairment. Moreover, the effects of the app emerged consistently and sustainably for >6 months. This offers great opportunities for implementation in the workplace, as the app has the potential to prevent burnout and reduce distress from work-related stress as well as decrease absenteeism.

## Abbreviations

BAT: Burnout Assessment Tool
CSES: Coping Self-Efficacy Scale
EMA: ecological momentary assessment
EMI: ecological momentary intervention
LMM: linear mixed model
PSM: preventive stress management
PSS: Perceived Stress Scale
RCT: randomized controlled trial
SCED: single-case experimental design

## Data Availability

The data sets generated during and analyzed during this study are available from the corresponding author on reasonable request.
